# The broad positive health perspective on health as common ground for tackling current and future interdisciplinary health challenges

**DOI:** 10.3389/fpubh.2025.1680808

**Published:** 2025-09-30

**Authors:** John A. J. Dierx, Cindy M. A. De Bot

**Affiliations:** ^1^Research Group Equal Chances on Healthy Choices, Centre of Expertise Perspective in Health, Avans University of Applied Science, Breda, Netherlands; ^2^Research Group Prevention in Nursing Care, Centre of Expertise Perspective in Health, Avans University of Applied Science, Breda, Netherlands

**Keywords:** broad health, positive health, patient centered, interdisciplinary collaboration, sustainable health care

## Abstract

In the past decades, many developments on economy, demography and technology amongst others have directly or indirectly influenced population health both in a positive and negative way. Consequently, the population is aging with accompanying demand for care while the workforce in health care and wellbeing is decreasing. A so-called infarction in health care and wellbeing is pending. This calls for a shift in perspective on health as care can be prevented by healthy lifestyle and resilience and self-reliance could be increased. Positive Health (PH) introduces such an innovative shift in healthcare, prioritizing resilience, adaptability, and overall well-being over traditional disease-focused models. This Perspective article explores the possible definitions of health and the development of PH, its pros and cons, application of the concept in the Netherlands and some examples worldwide and finally and discusses the global opportunities and challenges it poses. The article highlights future pathways toward a patient-centred and sustainable healthcare system, and it is suggested that the PH perspective could be instrumental in this emphasizing intersectoral collaboration, ethical considerations, and integration into healthcare frameworks.

## Introduction

Several developments economy, demography, and technology have influenced directly or indirectly population health (1, 2). Economic growth increased income which is associated with better health whereas it made the unhealthy lifestyle choice easier. Technological progress improved health care treatments, but computers and mobile phones have become more widespread, while physical activity has decreased, hence influencing lifestyle. These developments change demography by an increasing average age of the population and at the same time increasing chronic diseases with a corresponding increase in the need for care. Increased need of care while the number of healthcare professionals is declining (3–5). Therefore, a possible solution would be shifting focus from cure to prevention and promoting healthy lifestyles. Shifting from a disease and deficit perspective toward a salutogenic and assets-based perspective, with ‘assets’ defined as “any factor (or resource) which enhances the ability of individuals, groups, communities, populations, social systems and/or institutions to maintain and sustain health and well-being and to help to reduce health inequities” (6).

This paper calls for this focus shift and its challenges. Especially, emphasizing the role that a broad concept of health as Positive Health (PH) could play in this and in reducing the burden on the health care system.

## Redefining health

This change in perspective in health and its challenges requires revisiting the 1948 WHO health definition as “a state of complete physical, mental, and social well-being, and not just the absence of disease or infirmity” (7) implying that health is a static professional’s point of view on people’s health regardless of the perception of the individual and context. However, the aforementioned developments have not only changed professionals’ but also people’s views on health and attempts in redefining health to one more suitable to the times have been made (8–12). Core of the discussion is what parameters constitute the individual perception of health, what role contextual factors play in this perception and the fact that ‘health’ is not static but changing over time. Addressing these characteristics, Bircher redefined the original WHO definition adhering to Nordenfelt’s conception of health (9) being “a dynamic state of wellbeing characterized by a physical, mental and social potential, which satisfies the demands of a life commensurate with age, culture, and personal responsibility. If the potential is insufficient to satisfy these demands the state is disease.”

Where this definition clearly emphasizes the dynamic state of health and brings in the more contextual factors as culture and age, we believe it is still from an objective professionals’ perspective that is often deficit-based focusing on what is lacking with maximal achievable outcome. Huber et al. (2011) elaborated on this proposing a more ‘positive’ definition of health as “…the ability to adapt and self-manage, in the face of social, physical and emotional challenges” (13). This definition is dynamic, includes (social) contextual factors and is asset based from a patients’ perspective. Rooted in the salutogenic model developed by Aaron Antonovsky in 1979 (14, 15), it shifts the focus from the causes of disease to the origins of health, introducing the Sense of Coherence (SOC) emphasizing the ability to perceive life as comprehensible, manageable, and meaningful. It emphasizes the potential people have instead of what they lack and strives for *optimal* instead of *maximal* outcome. Consequently, the operationalized concept of PH added the three dimensions meaningfulness, quality of life and daily functioning to the dimensions bodily functions, mental functions, and social and societal participation of the WHO definition (16). This definition has met quite some critique, mostly on its wording but still very relevant (17–20). It was argued that it was too general comprising total life (20), the word ‘adapt’ was not specific enough (19) and the dimension quality of life is a part of health and not a factor determining health (19). However, citizens felt this definition aligned to their own perception of health, became more concious and felt more in control of their own health (21, 22).

## Examples in global contexts

The Netherlands has been a frontrunner in exploring the possibilities of PH implementation, providing insights into how intersectoral collaboration can address these challenges (23–25). From this exploration it appeared to be a potential model for other countries facing similar health system challenges. This can be illustrated by the examples in Dutch areas. First example is ‘Well in Flevoland’ which operationalized PH at a regional level, emphasizing professional training, lifestyle interventions, and social prescribing. The program reported a 20% reduction in hospital admissions over two years and a 15% increase in patient-reported wellbeing scores. Additionally, healthcare professionals noted a significant improvement in their ability to address social determinants of health, with 80% reporting higher job satisfaction (26). Second example is in Leidsche Rijn-Vleuten de Meern District where urban implementation focused on fostering neighbourhood cohesion and promoting lifestyle changes through initiatives like the Combined Lifestyle Intervention (CLI). Participants reduced their average weight by 12% and improved physical activity levels by 20% over six months. At the same time healthcare utilization decreased by 15%, highlighting the economic benefits of PH (26).

International examples on how PH’s is a relevant concept across diverse healthcare systems are also emerging. In Belgium integration of mental well-being into primary care includes targeted interventions such as stress management workshops and early detection of mental health issues, which align closely with PH’s emphasis on adaptability and resilience.^1^ In Colombia, PH principles are being applied to reduce disparities in access to healthcare by establishing community health programs that bridge urban and rural divides, leading to improvements in preventive care and vaccination rates (27). Iceland’s community-based mental health initiatives have successfully reduced suicide rates and increased access to support services, demonstrating the efficacy of PH in addressing societal challenges (28). Japan’s focus on older adults care has resulted in programs (e.g., Ikigai) encouraging active aging and social participation among seniors, directly by increasing resilience impacting their quality of life and reducing healthcare costs (29). Germany’s patient autonomy programs, e.g., personalized health plans for chronic disease management, showcase how PH principles can be tailored to enhance patient engagement and outcomes (30, 31). Despite these promising examples, the broader approach to health is not yet common practice. This is mostly because different professionals in health and wellbeing have different perceptions or interpretations of health and the concept of PH. The next paragraph elaborates on this based on our own research.

## Professional perspectives on facilitating and challenges of the positive health concept

In the past five years, we have studied the acceptance of the PH concept amongst a wide variety of professionals. The studies involved 693 professionals in healthcare and social work, including nurses, physiotherapists, and social workers (32–37). All studies used the same standardized survey for all professionals to complete followed by in-depth interviews of a representation (*N* = 8–10) of each group of professionals. The results of the qualitative thematically analysed (38) interviews are presented in this article.

## Total concept of PH

All professionals generally agreed that the total PH concept emphasizes strengths rather than weaknesses. This perspective was particularly strong among physiotherapists, who frequently mentioned that health is a dynamic state, emphasizing self-management and ownership. They believe that focusing on these aspects can empower patients to take control of their health. Hospital nurses highlighted the importance of recognizing that people are more than their diseases. They appreciated PH for its holistic approach, which includes self-management and the absence of hierarchy between patients and professionals. However, mental health nurses were less likely to mention these topics, indicating some variability in how different professionals within a group perceive PH.

*"Well, it sounds like a good model. What I immediately have to think about is working in a recovery-focused way. (...) So don't look at what you can't do but look at what you can do." (social worker)*

*"I think all these aspects are covered. One is a consequence of the other." (Physiotherapist)*

*"If you can deal with your disabilities or disease in a good way, to me that means someone is healthy." (hospital nurse)*

Despite these positive aspects, healthcare professionals as physiotherapists and hospital nurses, in contrast to those working in residential care like community nurses, youth care nurses, and social workers, questioned whether all patients/clients are capable of self-management. They also felt that not everyone wants to take ownership of their health. Another more negative aspect mentioned especially by social workers and mental health nurses, is that PH is a too broad concept and might be too time-consuming in consultations making it challenging to apply in practice. In particular nurses felt that the demands of their work often left little time to address all dimensions of PH.

*“While I see the value of addressing multiple dimensions of health, I often lack the time to delve into these areas during routine appointments.” (hospital nurse)*

*"The pressure of work means that there is not always enough time to look beyond what you have planned for the client." (homecare nurse)*

*"Within the acute phase of a mental health crisis admission, I wonder if Positive Health can be used as a method." (mental healthcare nurse)*

*“Self-management is a great concept, but many patients are not equipped to take full control of their health due to external circumstances.” (Physiotherapist)*

However, many participants were initially unfamiliar with PH in their daily work, and they recognized that elements of PH were already present in their practice.

*"We do everything. For me, Positive Health reflects the care you provide, your job. Actually, we all do it." (social worker)*

In addition, participants do not feel sufficiently competent in applying the concept and expressed a need for more practical interventions and knowledge about PH feeling that additional training and resources would help them better integrate PH into their daily practice.

*“Integrating these dimensions into my work feels overwhelming without proper training.” (social worker)*

*"I do need more knowledge and practical tools to be able to use this concept of positive health in my work." (youth healthcare nurse)*

## The six dimensions of PH

Physiotherapists, social workers, hospital nurses, and youth healthcare nurses all consistently prioritized quality of life as the most important health dimension. They regarded this dimension as an overarching concept that encompasses other dimensions of health.

*"Quality of Life. Physical and mental health problems can be as bad as they get, if you have certain things in life that make you happy and give you meaning in life and allow you to work towards your goals. Then I think your physical condition doesn't matter much." (homecare nurse)*

*"Quality of life is the most important one for me anyway. (...) I see the quality of life as a kind of umbrella for all the others*. If one dimension is not right, the quality of life will be greatly affected." *(social worker)*

However, mental health nurses and some physiotherapists and hospital nurses placed a higher importance on the bodily dimension, reflecting their focus on physical health.

*“…but I am very focused on these bodily functions in order to reassure them and to get a good picture of them." (physiotherapist)*

*"If you experience pain, for example, that affects every domain." (hospital nurse)*

Interestingly, there was considerable discussion about the dimension of meaningfulness. This dimension together with the dimension of mental wellbeing was deemed least important and if so, it was in combination with and related to other dimensions thus of least value in current practice. Nurses, particularly in home care, recognized the importance of both and despite time pressures tried to incorporate it into their work. However, they also felt that addressing meaningfulness might be more suited to social workers or psychologists. Especially physiotherapists, found it less relevant and struggled to find time to explore it with patients.

*"If necessary, we will have a cup of coffee or tea with a number of clients after the care moment to see how these clients are doing." (homecare nurse)*

Social participation and daily functioning were less emphasized in daily work but were acknowledged as important. Nurses also highlighted the impact of bodily functions on other dimensions, with pain being a significant factor affecting overall health.

*“I would perhaps set high standards for social participation and mental well-being myself if it were possible, but I am very focused on these bodily functions in order to reassure them and to get a good picture of them.” (physiotherapist).*

*"If you experience pain, for example, that affects every domain." (hospital nurse)*

*"Within the acute phase of a mental health crisis admission, I wonder if Positive Health can be used as a method." (mental healthcare nurse)*

## Discussion

Major developments in economy, demography and technology on population health (1, 2) call for a shift in focus from cure to prevention and promoting healthy lifestyles from a salutogenic and asset-based perspective. This paper presents an alternative perspective on the challenges in health and wellbeing of next decades especially highlighting the proposed role that the broad approach of health and more specific the concept of PH could play in prevention and consequently reduce the burden on the health care system.

PH represents a transformative framework for healthcare, complementing traditional disease management by addressing broader well-being factors. As promising as this might be from examples of the global contexts, our own data highlight both the strengths and challenges of implementing PH in healthcare and social work. While there is broad support for its holistic and empowering approach, professionals’ practical concerns about its applicability and the capacity for self-management remain. In doing so, professionals might underestimate the capacities of their clients who have applauded the definition and concept of PH reflecting their perception of health best (39). As preventive interventions have been shown most effective when aligned with client’s perspective, professionals should adhere to the concept creating more self-reliant clients and therefore reducing burden on health care in the future (40–44).

Overall, the findings suggest that PH is a promising concept, but its implementation requires careful consideration of the diverse perspectives and needs of different professional groups. The examples from the global contexts show that interprofessional collaboration is key in the successful implementation of the concept requiring a common conception and perspective on health and the PH-concept in particular. Some healthcare providers who use the concept report increased job satisfaction (41) and enhanced patient relationships due to the concept’s emphasis on multidimensional health. Encompassing physical, mental, and social domains, positions PH as a holistic approach that resonates with both patients and healthcare providers. Costa and Serra (2024) also recommended intersectoral collaboration by arguing that integration of disciplines is needed to tackle health issues (45).

The use of tools like the “spider web” facilitates meaningful patient-professional conversations and empowers patients to take an active role in their health (46). However, our studies show that this is not common practice. As mentioned by some professionals in our research, they need tailored training programs to equip them with the necessary skills and confidence to implement PH effectively (47). Furthermore, an interprofessional training program would help professionals use a shared language and, in that way, also facilitate interprofessional collaboration possibly contributing to improved outcome of treatment (46).

Regarding the dimensions of PH, all professionals perceived the emphasis on physical functioning and quality of life underscoring the need for a comprehensive approach to health that goes beyond mere physical well-being. Especially since this dimension aligns with the citizens’ perspective independent on age, ethnicity and socioeconomic position (39). This might imply that professionals addressing the quality of life in their conversation with clients, would be more successful in resonating with their clients’ perspective and increasing adherence to treatment or lifestyle advice resulting in less returning consults and hence reduced burden on health care. However, the lower importance placed on meaningfulness suggests an area for potential growth and development in professional practice. This might be even more important especially in palliative care (34).

The biggest concern mentioned by professionals is the costs that would increase as PH’s broader scope demands additional time and interdisciplinary collaboration, which can strain already burdened healthcare systems. However, by investing time and therefore financial resources in prevention and early detection, increasing self-reliance and through this less burden on health care, would not increase and even possibly reduce costs (48, 49). Furthermore, it could be argued that integrating PH principles into existing workflows could include development of digital technologies to streamline these processes reducing costs even further.

Summarizing, the PH concept represents a transformative framework for healthcare, complementing traditional disease management by addressing broader well-being factors. Operationalizing this concept through interdisciplinary collaboration, supportive health policies, prioritizing training programs, by allocating funding for pilot projects, and advocate for systemic changes that align with PH’s ethos is crucial for the healthcare sector to transition toward a more sustainable, patient-centred future.

## Future directions

To ensure the successful implementation of PH, several actionable steps and policies are proposed:

- *Enhancing intersectoral collaboration* by strengthening partnerships between healthcare providers, social services, educational institutions, and community organizations. Regular intersectoral meetings can establish shared goals and ensure smoother communication and appointing regional coordinators overseeing PH initiatives can facilitate alignment and resource distribution (26).
- *Expanding training and educational programs* to bridge the knowledge gap. Universities and professional bodies should integrate PH into curricula for professionals in health care and wellbeing including interactive workshops, real-life case studies, and role-playing scenarios and offering certification programs focused on its principles. Ongoing mentorship from experienced practitioners can further enhance the confidence of newly trained professionals.
- *Developing standardized metrics for evaluation* to address measurement challenges. Developing standardized metrics aligned with PH’s dimensions remains a research priority (50) focusing on further developing robust tools that capture PH’s multidimensional impact (51–53).
- *Developing technology and innovation* by designing digital health platforms, tools, and mobile applications for example digital dashboards for real-time monitoring and providing personalized health plans. These applications, controlled by patients, could also facilitate interdisciplinary patient centred communications and increase patients’ self-management.
- *Organizing policy advocacy* campaigns targeting stakeholders, including governments, health organizations and the public to increase support for PH. In addition, securing dedicated funding for pilot programs and scaling successful initiatives on the implementation of the PH concept through highlighting the long-term cost-effectiveness and societal benefits.

## Data Availability

The raw data supporting the conclusions of this article will be made available by the authors, without undue reservation.

## Footnotes

^1^ https://positivehealth-international.com/countries/belgium/
